# Intersectional analysis of the experiences of women who fail to conceive in low and middle income neighbourhoods of Delhi, India: Findings from a qualitative study

**DOI:** 10.1371/journal.pone.0304029

**Published:** 2024-07-03

**Authors:** Priyanka Adhikary, Gitau Mburu, Rita Kabra, Ndema Abu Habib, James Kiarie, Neeta Dhabhai, Ranadip Chowdhury, Sarmila Mazumder

**Affiliations:** 1 Society for Applied Studies, New Delhi, India; 2 UNDP-UNFPA-UNICEF-WHO-World Bank Special Programme of Research, Development and Research Training in Human Reproduction (HRP), Department of Sexual and Reproductive Health and Research, World Health Organization, Geneva, Switzerland; Makerere University, UGANDA

## Abstract

**Background:**

Experiences of delayed conception and infertility have been reported among women. However, the concept of intersectionality is rarely utilised in studies of infertility, and it is particularly uncommon in research from low- and middle- income countries.

**Research question:**

What are the lived experiences of women with delayed conception in low to -middle income neighbourhoods of Delhi, India?

**Methods:**

This was a qualitative study (n = 35) that recruited women who had failed to conceive after 18 months of regular unprotected sexual intercourse. Data were collected between February and July 2021. Data were collected through focus group discussions in low income to middle income neighbourhoods of Delhi, India. Analysis identified themes related to intersecting axes of inequality.

**Results:**

The results showed that gender intersected with economics, masculinity, patriarchal norms and class to influence the experiences of women. The intersection of gender, economics and patriarchal norms compromised women’s agency to be active generators of family income, and this dynamic was exacerbated by patrilocal residence. In addition, masculinity contributed to stigmatisation and blaming of women, due to the inaccurate perception that men did not contribute to a couple’s infertility. The intersection of gender and social class in medical settings created barriers to women’s access to medical information.

**Conclusion:**

Findings from this study provide representative examples of the variety of axes of inequality that shape women’s experiences in the study setting. Although these findings may not be generalisable to all women who are experiencing delayed conception, they highlight a need for improved awareness and education on infertility, as well as a need to ensure the availability and accessibility of fertility care for couples in need.

## Introduction

Infertility is an important public health issue globally [1]. It is defined as a disease of the male or female reproductive system, characterised by the failure to achieve pregnancy after 12 months or more of unprotected sexual intercourse [2]. Infertility as a public health issue is closely linked to biological sex because it is concerned with bodily reproductive function [3], as well as gender and gender roles [4]. As a social construct, gender is what people are expected to do in their everyday lives, based on their biological attributes [5]. Consequently, gender is shaped by social interactions [4, 5] within a wider structural environment [6]. As such, an exploration of how infertility is experienced by women or men can reveal how gender is linked with structural drivers of gender-based inequalities [6].

In several low and middle income countries (LMICs), infertility has significant implications for women. A woman’s ability to beget a child determines her identity and womanhood. The kinship system, together with patriarchal descent, patrilocal residence, and property inheritance, all contribute to the value of fertility intertwined with women’s status in such societies [7, 8].The normative constructs of femininity and masculinity reinforce man’s role as a protector and breadwinner, while the woman is expected to be the keeper of the house and raise children. In these contexts gender roles endorse a woman’s primary role as a mother [9]. Only in becoming a mother does she fulfil her role in society. Deviating from normative gender expectations surrounding motherhood is discouraged by mainstream society, and often results in stigmatisation [10–13].

In this context, we posit that intersectionality is a useful concept for elaborating women’s experiences of infertility. Such experiences may reflect diverse intersecting factors such as age, class, ethnicity, education and financial capacity, since these and other factors affect women’s social positions and influence the multiple interactions of their social identities [14, 15]. An intersectional approach therefore has the potential to illuminate multiple dimensions of women’s lives that co-exist and interact to shape their lives [16, 17].

Although the concept of intersectionality is increasingly used to understand inequalities [16], it is rarely employed in infertility studies [15]. An intersectional analysis of infertility experience is useful as it provides an overarching view of how various social facets act as instruments in shaping women’s experiential journey of failed conception, and consequently, how these facets contribute to gender inequality [15]. This approach is particularly useful in elaborating context-specific interactions of social categories and the power dynamics that produce or uphold inequalities [18]. In this paper, we draw on an intersectional approach to provide insight into the lived experiences of women who failed to conceive in low to middle income neighbourhoods of Delhi, India. The paper explores the drivers of inequality among these women using an intersectionality approach. Findings from this study will provide useful information that can be used to improve health services and social support for women who failed to conceive a child.

## Materials and methods

### Study aims and design

This was an exploratory qualitative study conducted among 35 women who had failed to achieve pregnancy with their husbands/partners after 18 months of regular unprotected sexual intercourse, as reported by them. Qualitative methods are suited for exploration of people’s experiences [19]. The study was nested in a larger cross-sectional mixed methods study that aimed to understand (a) the baseline characteristics of women who experience delayed conception; (b) their perspectives, actions and experiences related to the delay in conceiving; (c) their quality of life and mental health; and (d) costs of any interventions obtained. The study was carried out in urban and peri-urban low-to-middle socio-economic neighbourhoods of South Delhi, India [20]. Study sites were selected based on feasibility, given that investigators had previously been running a randomised controlled trial in the area [21] and had built a rapport with community leaders. Data were collected over a period of 6 months between February and July 2021. Reporting in this paper follows COREQ guidelines for qualitative research [22] as shown in S1 Checklist.

### Participant recruitment

A detailed protocol for this study has been published elsewhere [23]. In brief, participants were selected from the population of women who had participated in the Women and Infants Integrated Interventions for Growth Study (WINGS) [21] and had failed to become pregnant during the 18-month study follow-up period. WINGS was an individually randomised trial with factorial design that assessed the impact of an intervention package comprising nutrition, health, psychosocial, and water, sanitation and hygiene (WaSH) interventions spanning across the preconception, pregnancy and early childhood period [21].

At the end of the 18-month study follow-up period, women who had failed to achieve pregnancy with their husbands/ partners were invited to participate in this study on infertility. A total of 1530 women had failed to achieve pregnancy with their husbands/ partners in WINGS despite being sexually active, not using contraception, and not lactating over a period of 18 months. Of these, 775 had primary infertility (i.e., had never conceived), while 755 had secondary infertility (i.e., had conceived previously). Among these 1530 women, 35 were purposively sampled to participate in qualitative focus group discussions (FGDs), until saturation of data. Through this sampling method, researchers identified individuals who had never been pregnant (i.e., had primary infertility) as well as those who had a child and wanted one more child (i.e., likely to have had secondary infertility), in order to ensure the inclusion of women with diverse experiences. Participants were contacted by researchers via a combination of phone calls and in-person by visiting their homes.

### Study sample

Thirty five women who had failed to conceive over a period of 18 months constituted the sample for this study. Table 1 presents the demographic characteristics of the participants, who were identified using a numerical code to protect their identities. Of the 35 women, nine already had a child and wished for a second child. Most of the participants were Hindu (n = 28), while others were Muslim (n = 4), Christian (n = 2) and Jain (n = 1). The mean age of participants was 27.68 (± 3.25 SD) years, with an age range of 21 to 33 years. All were married and identified as heterosexual; 33 were homemakers, one participant was working in the private sector and one was self-employed, managing a grocery store. The average annual household income was 220,000 Indian Rupees (2674 USD). Only two of 35 had health insurance.

**Table 1:** Characteristics of study participants (N = 35).

Social demographic characteristic		Proportion (number)
Age	Mean age	27.68
	Age range	21–33
Education	Illiterate	8.57% (3)
	Elementary education	65.71% (23)
	Undergraduate	22.85% (8)
	Postgraduate	2.85% (1)
Occupation	Homemaker	94.28% (33)
	Employed	5.71% (2)
Sexual orientation	Heterosexual	100%
Religion	Hindu	77.14% (27)
	Muslim	14.28% (5)
	Christian	5.71% (2)
	Jain	2.8% (1)
Health insurance	Had health insurance	5.71% (2)
Caste	General	34.28% (12)
	Scheduled	42.85% (15)
	Minority	22.85% (8)

### Informed consent procedures

Ethical approval for this study was obtained from the Ethics Review Committees of the WHO (A-ID: A65998) and CHRD-SAS (SAS/ERC/RHR-Infertility/2020). Participants were informed about the aim of the study, study procedures, voluntary participation denoting that they were entitled to decline participation or withdraw from the FGDs, or decline to respond to questions at any time, even after initially consenting to participate. Those who consented to participate were enrolled. The FGDs were recorded if the women consented for the same. Participants were provided with a unique number, with which they could be identified, and their personal information was anonymized and their identity was kept confidential. FGDs were conducted by a female researcher (PA) who is trained in qualitative research. Before each FGD, the researcher confirmed that participants understood the purpose, benefits, and risks of participating, and then obtained their written consent to participate. This was done to ensure informed decision-making to participate.

### Data collection procedures

To guide the FGDs, topic guides were prepared. The topic guide questions focused on the experience of delayed conception, coping mechanisms, treatment-seeking behaviour, and the type of help sought, including from family, friends and medical professionals, and other actions taken by the participants to try to increase the likelihood of conception. The topic guide was designed to explore areas where the participants struggled with during delayed conception, and about decisions that participants made in their efforts to conceive.

Prior to data collection, the topic guide was translated into Hindi script, the local vernacular, and pilot tested on a small sample of women (n = 6). The topic guide was then refined with the aims of making questions more context-specific, ensuring that questions would be relevant to participants, and would elicit meaningful responses, while maintaining sensitivity to the challenges associated with discussing infertility, such as stigma.

Following this, four FGDs were conducted with 12 participants (First FGD), 8 participants (Second FGD), 8 participants (Third FGD) and 7 participants (Fourth FGD). The FGDs were conducted in Hindi.

Although the FGD moderator i.e. the researcher and the participants shared a common socio-cultural background i.e. nationality, language, and gender, the moderator was cognizant of her socio-cultural privilege including education, status, and class. She was aware of her situatedness in the study and therefore constantly negotiated her positionality as a researcher and as an individual who has her own subjective stance, shaped by her personal experiences, socio-cultural status, and surroundings. During the discussions, the researcher reflected on her objectivity as a researcher, and her subjectivity as a human being sharing a social niche that was different to that of the participants. What was paramount for the researcher was to make the women feel comfortable. She attempted to remain empathetic and sensitive towards the participants, while accepting her positionality. In order to make participants comfortable and not self-conscious, the researcher dressed similarly to the women and went along with the conversation, switching languages between Hindi and English when participants did so, and with humour, attempted to put them at ease.

The FGDs were conducted at a neutral place outside the home environment, not in any clinic that can be identified with infertility care. This was to avoid apprehensions and safeguard privacy and confidentiality, while enabling participants to feel comfortable while interacting with the researcher. The FGDs were carried out in the recommended seating arrangement, in a circle with a distance that was appropriate for each one to be clearly visible and heard, which facilitated engaging discussions. Multiple dictaphones were placed at various positions to capture the voice of all participants. Along with field notes, participants’ non-verbal communications were documented to obtain deeper insights into their responses. The FGD sessions lasted for an average of 60 to 90 minutes. At the end of each FGD session, the topic guide was cross-checked to confirm that all questions had been asked. The key points were shared with the participants for their final comments and to ensure that their perspectives were appropriately captured.

### Data analysis

The lead researcher who conducted the FGDs (PA) transcribed the recordings and was involved in data coding. By performing each transcription shortly after the FGD took place, the researcher was able to detect and recall nuances in the responses and observations, thus maximising the accuracy, reliability and completeness of the data. Given the complexity of translation [24], the research group selected a qualitative researcher (PA) who was fluent in writing, reading and speaking Hindi language, which preserved participants’ meanings and trustworthiness of the translation. Where Hindi words could be translated into several closely linked English words, the researcher strived to take into account the context of the discussion, participants’ words and gestures in order to mitigate the risk of loss of meaning during translation.

The transcripts were analysed using NVIVO software, version 1.7.1. First, similar responses were coded and grouped in nodes, which were then used to generate sub-themes and themes through an inductive thematic process [25]. Patterns and relationships among the themes and sub-themes were identified and conceptualized iteratively to ensure that the potential significance of all the data was fully explored. A code book was generated by PA text by text, which identified themes and sub-themes, and codes, corroborating the theory of intersectionality. The generation of the codebook was an iterative process in which each text was read repeatedly, followed by generation of themes through a combination of both inductive (data-driven) and deductive (theory of intersectionality) approach [26]. The lead researcher (PA), who is a social scientist with doctoral level training in anthropology, and was unknown to the participants prior to the study, was aware of the potential for her own background, values and beliefs to influence data collection and interpretation of research findings [27]. The researcher (PA) initially coded the data, and discussed the preliminary findings with the larger research team comprising all the authors, and modified codes following discussions. To further mitigate researcher bias, the analysis hinged on verbatim and bodily expressions of the study participants to capture the richness and depth of participants’ experiences.

## Results

The results provided insight into how gender intersected with economics, masculinity, patrilocal norms and class to influence the experiences of women, as shown in Table 2.

**Table 2:** Intersecting axes based on emerging themes.

Axis	Description of thematic codes
Gender and gender roles	Gender norms as prescribed for women and men; expected gender roles
Masculinity-related behaviours and patrilocal norms	Norms related to how men behave or should behave Norms that prioritize patriarchal practices, e.g., patrilocal residence of couples
Social economics	Financial means and ability; source of income; labour participation Social class based on background, education and profession

### Intersection of gender, finances, and labour participation

The socioeconomic status of many study participants and their families meant that the cost of infertility treatment imposed a major burden. In many cases, couples made financial sacrifices or were forced to forego other needs in order to pay for it:

*We*, *my husband and I*, *have prioritised the treatment and medicines required to have a child rather than spending money on food*. *We have cut down our budget just for the treatment*.

(*FGD participant* # *28*)

Having experienced high treatment costs, many women understood the financial difficulties they and their husbands faced. The women shared that they performed their duties in the household as per the gender norms with all dedication. They believed that being committed to the desired gender specific duties in the household will project their image as a good and responsible daughter-in-law who contributes her labour efficiently in the household. Therefore, they felt by doing so, they would not be taken as a burden to the family members. Nevertheless, despite all their efforts their failure to conceive a child devalued their role and duties in the family and they were perceived as an economic liability by their in-laws:

*My mother-in-law’s behaviour fills me with immense pain because of her disdained comments [such as] “What good is she for if she cannot bear a child*, *but never forgets to eat food on time*.*” It has been harrowing for me to experience such negativity from her*. *Even though I try to be engaged in household chores and stay in my room*, *her words eat me up from inside*.

(*FGD participant # 7*)

The link was commonly made between economic consumption and women’s inability to contribute to the household by bearing children. This link was a catalyst for feelings of shame and internalised stigma:

*Since my husband’s family is spending money on me with the hope of me conceiving a child*, *I hesitate to ask for anything extra if I am in need of it*, *or if I want to hang out with my cousins*. *I struggle with feelings of guilt for using up their money for my treatment*.

(*FGD participant* # *4*)

The idea that women were economically draining their families without producing an offspring was a common reason for overt stigmatisation by family members, particularly in the context of patrilocal residence, where women were observed by other family members on an ongoing basis. Women’s consumption of food and other household necessities was considered a waste of money since these women did not reciprocate by bringing a child into the family:

*I admit that my husband’s family has spent way more than I could imagine*. *I sought treatment from both private and public health facilities*. *I have visited 15 doctors to date*. *Whatever we earned*, *we used it on my treatment*. *My mother-in-law often taunts that I brought bad luck to the family with my unsuccessful pregnancy attempts*. *She also taunts me with the thought that I earn just enough to pay for the medicine and treatment*. *She provokes my husband by saying*, “*Your wife will never get pregnant no matter how much money you waste on her*.” *To date*, *about 5–7 lakhs [equivalent to USD 6100–8600] have been spent on my treatment*.

(*FGD participant* # *22*)

Faced with high treatment costs and perceptions of only being consumers in the household economy, many women were keen to contribute to paying for the cost of their infertility treatment by earning money. However, they reported being discouraged from working and told that they should stay in the homemaker role:

*I am a housewife and I do not think I will be allowed [by the husband’s family] to get a job anywhere*. *Neither my parents nor my husband’s parents will like this idea*. *If I had this opportunity to work*, *I would not have to be disdained by my husband*, *who thinks he is wasting his money as he sees no positive outcome of pregnancy*.

(*FGD participant* # *18*)

Other women reported being actively discouraged from seeking employment or other forms of labour participation. Financial freedom seemed to have been confiscated for these women. It was not unusual to hear that “*my mother-in-law disallowed me from finding a job*,” as stated by one participant (FGD participant # 9). It was clear that these women’s financial ability was greatly limited as their families sought to conform to social expectations. FGD participants supported the idea that in accordance with the prevailing gender roles, a woman was to stay at home and be a homemaker; she was expected to allow the husband to provide economically, and to depend on him:

*I have reached that stage of my life that I have spent all we have on treatment*. *But my in-laws have a problem with me taking up a job outside*. *I have to ask people to lend me money for treatment*.

*(FGD participant # 2*)

When women could not access treatment because their husbands were unemployed and were unable to fulfil the prescribed role of provider, this contributed to tension between the expected gender roles and the perceived economic implications of infertility:

*My husband was jobless for some time*, *and I could not seek medical assistance due to financial strain*. *After he found a job after months*, *my mother-in-law and sister-in-law did not like him giving me money to see a doctor*. *I heard her say*, “*Do not spend your hard-earned money on your wife*. *She will not bring a new member into the family*.”

(*FGD participant* # *11*)

According to the FGDs, it was considered that a woman would bring dishonour to the family by working. As one participant noted, *being a daughter-in-law*, *I am responsible for keeping the family’s honour*. (FGD participant # 6) Consequently, the husbands’ family members disapproved of the idea of a daughter-in-law seeking work outside of the home. By seeking employment, it was perceived that women would be exposing their husbands as unable to meet the needs of their families. Participants explained that the husband’s kin, i.e., the woman’s brothers-in-law and mother-in-law, found it shameful for a family to send a daughter-in-law to work. Doing so was thought to damage the pride of the family in the eyes of the husband’s kin, neighbours and society at large:

*Due to societal pressure*, *my mother-in-law always talks about losing the dignity of the house if a daughter-in-law goes out for work*. *She thinks our family will be criticised by the neighbours for letting me take up a job*. *She feels that she will be the face of embarrassment and will be seen as someone who uses her daughter-in-law as a financial provider of the family*.

(*FGD participant # 30*)

As a result, most study participants did not pursue the opportunity to work. Two women in the study sample worked and contributed to their treatment, but it was explained that this was frowned upon:

*My husband did contribute towards treatment costs as well*, *but that is not the point*. *I too have spent so much*. *But despite giving all I have got*, *my mother-in-law’s outrage never stops*. *She berates her son; my husband*, *for allowing me to pay for my medical expenditure*. *Can you fathom that [it is expected that] I should not spend money on my treatment*, *treatment that can allegedly help restore family dignity in the eyes of outsiders*.

(*FGD participant # 33*)

Thus, study participants’ autonomy in the labour market was curtailed in order to uphold the gender roles of women as homemakers and husbands as breadwinners, even though this undermined their ability to pay for medical services that could potentially enable them to fulfil expectations about childbearing. Challenging their prescribed roles led to negative consequences:

*Initially*, *my husband did not allow me to take up the job as it was against their family values where the daughter-in-law is supposed to stay inside the home and look after her husband and his family*. *He continued to object to my decision for about a month*. *In fact*, *he got too far to raise his hand on me*. *He slapped me*. *But I was determined and did not give up on my decision to take up a job*. *I got a job*.

(*FGD participant # 34*)

It should be noted that as opposed to the woman quoted here, who pressed on and acquired a job, almost all the other study participants were dependent on their husbands. Not surprisingly, many ended up borrowing money from their own parents or friends:

*My brother-in-law and my husband’s parents never liked that my husband spent on doctors*, *check-ups*, *and medicines*. *So*, *I tried to find an alternative way to cover the medical expenses and asked my mother to lend me money*.

(*FGD participant # 23*)

However, borrowing money for treatment, sometimes in secret, imposed the burden of indebtedness:

*My parents sent me money for treatment*. *They are still sending me money*, *hoping for me to become pregnant*. *I owe them a huge debt*. *Sometimes my father makes me feel that I was being born a burden to my parents*. *He does not talk much with me*. *It is only my mother who has been supporting me all along*.

(*FGD participant # 10*)

Across the study sample, findings showed that finances were a problem, even when study participants sought assistance from parents and friends. One woman said, *She [my mother] did [lend me money] at first*, *but later could not as she herself was in dire straits*. *Her hands were tied financially*. *(FGD participant # 15)*. Thus, some of these women were left in a dilemma whereby they could no longer borrow, nor work to pay for treatment.

### Intersection of gender, economics and masculinity

In this study population, the role of gender was also apparent with regard to how men’s potential contribution to infertility was understood. In nearly all cases, women were blamed for failing to conceive, even in cases in which men had not undergone fertility assessments:

*Everybody points at the woman*. *Nobody realises that one can’t clap with just one hand* (i.e. it takes two to tango). *People behave as if men are born fertile*.

(*FGD participant # 20*)

A consistent finding was that men did not visit physicians to have fertility assessments performed:

*He would not go to any clinic no matter how many times he is told*. *I know if he did*, *then his manliness would be put under question*. *He is so firm that the problem lies with me*.

(*FGD participant # 5*)

This is particularly notable given that the initial tests for men were thought by the participants to be relatively cheaper compared to infertility evaluations for women:

*My husband and I both went through diagnoses*. *Surprisingly*, *his test was less expensive than what I had anticipated and not unaffordable in comparison to ours as in women’s*. *In my case*, *I had to go through multiple tests while only one test was prescribed for him*, *which was [a] test of his semen*. *We ended up paying more for my test than for his*.

(*FGD participant # 29*)

In several cases, husbands either backed away from taking their wives to health clinics or reneged from doing so. A participant mentioned that *what is ironic is that when it comes to partaking in escorting me to the hospital or a clinic*, *I see none of my family members are ready to take me*. *(FGD participant # 25)* In addition, in-laws were unhappy that husbands spent money on what was essentially perceived to be a woman’s fault. As a result, husbands often were reluctant to pay for women’s treatment costs, and often, they were explicitly discouraged from paying by their families:

*My husband’s parents have forbidden my husband to pay for my treatment*. *His parents pick at me and hold me responsible*.

(*FGD participant # 17*)

In several cases, apart from having their husbands being actively discouraged from paying for their treatments, women were also blamed for “wasting” their husbands’ earnings, given that they had failed to conceive despite visits to medical practitioners:

*I have been married for about 15 years now*. *The reason my mother-in-law used to beat me up was because I could not give them what I was supposed to give them*: *a child*. *Moreover*, *she could not put up with the fact that I am the reason my husband frittered away his earnings and yet I remained childless for so long*. *Now we are not on talking terms*. *Yet*, *I am responsible for keeping the family’s honour*. *I often wonder how righteous it is to beat the one who is the family’s face of dignity*.

(*FGD participant # 21*)

It was clear that the patrilocal organisation of families was not generally conducive to or supportive of these women, as explained by one participant who stated that she ended up convincing her husband to stop living with her in-laws:

*I was emotionally drained and hemmed in by my husband initially when I tried getting him to move us out of the home*, *[and we eventually] started living on our own*.

(*FGD participant # 32*)

### Intersection of gender and social economic class in the context of medical treatment

The intersection of socio-economic class and gender during consultations with physicians shaped the experiences of study participants in multiple ways. Findings illustrated how differences in power and status were underlined by class and gender:

*The doctor said that because I am 33*, *I am not getting pregnant*. *He was like*, *“Why are you so late to visit my clinic*? *Do you women not understand the effect of ageing on fertility*? *Now it is difficult for you to get pregnant*.”

(*FGD participant # 1*)

Interactions of this nature, which were commonly reported, demonstrated how physicians serve as having gender bias and lack professional ethics in this study community, yet they are the gatekeepers to information that can facilitate or limit access to infertility treatment. Several accounts indicated that study participants, keen to pursue treatment that would redeem their identity and self-esteem, encountered stigmatization by some doctors in the study community, who generally attend to middle-class families. Many of the women in our sample did not have medical insurance, and they held multiple subordinate group identities as unemployed low-income women, mostly from scheduled castes and other backward castes. They did not seem to be aware of the schemes such as Pradhan Mantri Jan Arogya Yojana (PM-JAY) and other Ayushman Bharat Schemes. Some women reported experiences of being negatively judged in medical settings:

*I asked about the reason for my failed pregnancy when the doctor checked my test report at the hospital*. *He replied with a condescending voice that there is a lump in my uterus*. *I did not understand and asked him for an explanation*. *To which he responded*, “*You would not understand even if I explained it to you*.” *I later learned this is called a fibroid*.

(*FGD participant # 27*)

As the above verbatim indicates, doctors made assumptions about the participants’ ability to grasp basic information about their reproductive organs or their reproductive potential, which ultimately exacerbated their experiences of marginalisation in medical settings.

While these experiences were not always negative, the only positive experiences that were reported involved female physicians, as illustrated by the following excerpt from one response, a participant who had visited several physicians:

*I have gone to about five doctors so far*. *All of them prescribed ultrasound and other tests*. *One of them was nice to me*. *She would listen to me and console me because I happened to break into tears as I was mentally exhausted with drifting door to door and changing doctors*. *I felt that she understood what I was going through and gave me hope*, *which otherwise is rare these days*. *Not only did she give me hope but listened to me which never really happens with the [other] doctors*. *They are always in a hurry*. *I remember once a doctor did not give me time to listen to me and not even five minutes he interacted*. *He just prescribed a few medicines and a test and was done with me*.

(*FGD participant # 8*)

A similarly positive experience was reported by another study participant who was attended by a female physician:

*The doctor I had gone to assured me that I could get pregnant once my thyroid treatment is done and resolved*. *When I was getting a negative attitude from everybody from family to friends*, *she was the one who was the beacon of hope*.

(*FGD participant # 14*)

In sum, participants’ experiences within medical settings may have been impacted by the intersections of gender and class and its attendant social privilege.

## Discussion

This paper demonstrates how infertility reinforces gender inequality, through the intersection of gender norms, economics of treatment, and labour participation; produce experiences of marginalisation and dependency among women of low to middle socio-economic status in our study community. This is an important contribution given that despite the potential for the convergence of many determinants of inequality, the concept of intersectionality is rarely utilised in studies of infertility [15], particularly in low- and middle-income countries [16].

We demonstrate how among women who were trying to access costly fertility treatment, the intersection of gender, economics, and class excluded them from labour participation, marginalized them financially, and limited their access to medical information. These experiences were further exacerbated by the gendered role and masculinity of husbands. In this context, failing to enact motherhood as per societal expectations reinforced gender-based discrimination, which was further exacerbated by the economics of paying the high cost of fertility treatment.

Patrilocal family structure has long been ingrained in Indian society [28]. Women inhabiting patrilocal systems are less likely to have agency in areas including mobility and healthcare decisions than those living in nuclear households [28]. Furthermore, the interaction between family structure and participants’ experience of infertility was made more apparent by the patrilocal residence of participants, whereby newly married women live with their husbands’ families. In this situation, a woman’s identity automatically shifts from being a daughter of her natal family to the daughter-in-law in another household [29]. This habitation provides the grounds for the perpetuation of the dominant social discourse that promotes the idea that women’s primary role is motherhood. The intersection of economics and gender within patrilocal residence also provides the grounds for the paradoxical observation that while men were expected to be providers, women’s responsibility for producing children was not expected to impose any extra economic burden on their husbands. The influence of in-laws in upholding these expectations is not surprising given that while the study participants shared the same household with their-in-laws, they occupied a subordinate position [30], which undermined their ability to act on their choices and decisions [31, 32].

Feminist scholars theorise that the basis of women’s subordination is largely economic, given that women who are confined to the domestic space become economically dependent on men, which thereby, provides the opportunity for oppression. All except two of the study participants were homemakers and were entirely financially dependent on their husbands to access healthcare. Participants’ decision-making autonomy in relation to their reproductive health was therefore subjected to hegemonic family norms that excluded them from obtaining the financial means to access medical services.

In several communities, the rationale for discouraging married women from taking up work is to protect the family honour, which places the onus of giving the family a good name upon married women [33]. Women’s mobility and activity are therefore controlled to some extent [30, 34]. Any deviation from this norm may disparage a woman’s moral character, and have implications on economics and their ability to redeem their identity as women in the face of infertility and costly infertility treatment. Our study findings suggest that family prestige is prioritised over addressing women’s needs in relation to infertility. The participants in our study were unable to obtain financial means to pay for medical assistance.

Findings also suggest that gender socialisation shapes the expectations that traditionally assign women to the role of having a biological obligation to bear children in the study setting. When these gendered expectations were not met in our study population, infertility was perceived as a woman’s problem, similar to observations made elsewhere [35]. Thus, women were blamed on account of failing to mother a child, while men were not equally held responsible. Furthermore, this socialisation promotes the notion that motherhood is a defining factor of womanhood and that it is the only way to be fulfilled as a woman, as perceived in some communities. Other scholars posit that Indian women acquire respect, honour and power in a household by virtue of being mothers and homemakers [36]. Yet the economic marginalisation of women who are unable to attain pregnancy is a deterrent to women’s ability to achieve this status.

Examining the intersection of gender, economics and patriarchal norms enabled us to identify how patrilocal residence was associated with women’s experiences, as this played a part in compromising women’s agency as generators of family income. Although economic reasons have been identified as a motivating reason for patrilocal residency [37], we also show that it can have a negative impact on women labor participation. We noted that some of our participants’ family members discouraged them from entering the labour market, a finding that is consistent with the expectation of women being subservient, compliant, passive, and docile and attached to the home [38]. Such gender doctrine validates traditional gender roles, which set a precedent for female subordination in which women are denied autonomy, which subsequently leaves them with limited control over their lives. Not only did patriarchal structures obstruct women from enjoying the right to make decisions, but the exertion of patriarchal values by mothers-in-law exacerbated their subordinate position in the family. Along with various forms of domination, controlling women’s financial autonomy in the family unit was one of them. Male household members and in most cases participants’ husbands were the earners, while women were hamstrung by the supremacy of mothers-in-law. Similar observations, including the impact of work participation on women’s agency, as well as the detrimental effect of patrilocal residence on the women’s share of the labour market, have been made by other researchers [33, 39].

The intersection of gender and masculine norms allowed us to acquire an understanding of the ways in which masculinity can contribute to women’s experiences in relation to infertility, including through the perceived notion that men do not contribute to a couple’s infertility. Studies suggest that fear of being emasculated at the hand of society leads men to avoid being assessed for their fertility [3]. This shapes how women are solely held responsible for conceiving. Participants in our study reported that most of the male partners who were asked to undergo fertility assessment refused to do so. As described by the women, their husbands equated attending a fertility clinic with experiencing a direct attack on their masculinity, which is also consistent with observations that both virility and fertility are often linked to manhood [40].

Our study findings indicate that the intersection of gender and social class in medical settings has the potential to further shape women’s experiences in relation to infertility. Although the impact of class has been explored among women with infertility, for example in Bangladesh [14], the exploration within medical settings is rare in low- and middle-income countries. In our study two participants perceived that their status may have been a barrier in receiving appropriate care. Although this was not observed commonly in our study setting, discomfort and mistreatment in medical settings has been reported in high income settings [15].

### Implications for service and policy

These findings have several implications. First, findings suggest that there is a need for comprehensive information in the study setting to raise awareness about infertility, increase understanding about its causes, enhance knowledge about the various government insurance schemes, and ensure that the population understands that there are infertility treatments that can be accessed by couples. It is important for the general population to know that infertility can result from female factors, male factors, a combination of female and male factors, and in some cases, unexplained factors. In the study context for example, Accredited Social Health Activists (ASHAs), who are a cadre of community health workers, can be trained to include infertility as part of the information they routinely provide to communities.

From a social perspective, there is a need to find ways to provide women with access to peer support groups that can mitigate the negative psychological experiences of infertility. This proposal is consistent with other researchers’ calls for interventions to address the stigmatisation of infertility [9, 41]. Here, Anganwadi Centres (a type of child care centre in India) could play a role, and community health workers including ASHAs could be trained to facilitate such peer support groups for people with infertility. Additionally, given that stigma and discrimination is linked to gender norms, roles and expectations, it is critical to implement gender transformative interventions through behaviour change communication to sensitise communities on harmful gender norms and de-stigmatise infertility.

In terms of health systems, health care providers in medical facilities should provide counselling to women and couples who are dealing with infertility. Most counselling interventions are often low-cost and can have a significant impact on how infertility is experienced in the short term [42]. Furthermore, healthcare providers need training and sensitisation regarding infertility, and the need to listen to, respect and communicate in a non-judgmental way to patients with infertility. In the longer term, however, it is critical for governments and other stakeholders to aim for universal access to fertility care, including assisted reproduction technology (ART), as part of universal health coverage. This is particularly important given that private sector provision, and lack of regulations have been associated with higher costs of fertility treatments in low and middle income countries [43].

### Implications for future research

Our study adds to the rare literature on the gendered nature of how infertility is experienced and shows that individual experiences are shaped by an intersection of multiple social factors. In response to the need to explore circumstances that may exacerbate some experiences and identities more than others, we have demonstrated how the patrilocal residence can exacerbate women’s struggles relating to infertility. We suggest that women’s experiences might differ if living arrangements were separate, and this hypothesis could inform further research in this area. Further research could explore intersections of other axes including education, income levels, migrant status, and religion among others. In addition, more in-depth research is needed on how to scale up the presently available fertility care within the government health systems in order to further improve women’s positive experiences and empower couples to receive these services.

### Study limitations

This study had several limitations. Women who participated in the study were part of a pre-conception randomized (WINGS) study where they had been followed up to 18 months and failed to achieve pregnancy during this period. Recruitment was based on women’s history of failing to conceive over an 18 months period, despite intentions to achieve pregnancy with their partners. The study intended to understand the perspectives and experiences of women, but many of them did not go through any clinical diagnosis to confirm infertility. Therefore, while the study captured the experiences of these women it does not confirm a diagnosis of that infertility. Delay or failure to conceive affects a couple; however, only women were recruited in this study and they were asked to report what partner’s attitude and reactions were. Perspectives of male partners would have been useful, particularly because women tend to be blamed when male factors can also contribute to infertility. Future studies will need to include men.

Although intersectionality was applied to gender and economic axes of inequality, other factors not identified in this analysis may be at play among the study sample. Failing to identify complex dimensions during analysis is common even when multiple axes are explicitly examined together [44]. However, we chose to interrogate the dynamics of gender and financial status given the gendered view of biological reproduction and the high cost of infertility treatment in most low- and middle-income settings [43].

Our sample entirely comprised women of low to middle economic status, we therefore applied an intra-categorical approach to intersectionality analysis [45], and showed how economics and masculinity affected women’s experiences. Our intention was not to compare men’s and women’s negative experiences, which is postulated to be milder among men [46], but to explore how masculine norms affected women’s experiences. Expanding the analytical focus to encompass different genders, economic levels, castes and other identities could be useful in future research. Although extensive thematic analysis was conducted through the aid of software, it is possible that some relevant themes may remain unidentified [25]. Although reflexivity seemingly neutralised the emic (insider) and etic (outsider) perspectives of the lead researcher (PA), it is possible that her positionality could not be entirely discounted [47]. Although attempts were made to limit the extent to which the research team’s backgrounds, values and beliefs impacted on the data collection, interpretation and reporting of results it is impossible to entirely eliminate these influences [27].

## Conclusion

Infertility is often an invisible problem and poor unemployed women are often an invisible population. This study provides needed attention to a neglected topic in global health by examining the experiences of a marginalized group of women who have been unable to conceive children with their husbands. Through the application of intersectional analysis, this study shows how gender, economics and masculinity intersect to influence women’s experiences of this problem. Findings demonstrate the need for improved awareness and education on infertility and highlight the need for additional studies applying intersectionality to better understand perspectives of women, and the need to improve counselling and other infertility interventions for women and their spouses. These findings support calls to ensure that infertility is given more attention and that fertility care is available for all.

## Supporting information

S1 Checklist COREQ (COnsolidated criteria for REporting Qualitative research) checklist. (DOCX)
